# Leptin regulates disc cartilage endplate degeneration and ossification through activation of the MAPK‐ERK signalling pathway *in vivo* and *in vitro*

**DOI:** 10.1111/jcmm.13398

**Published:** 2018-01-26

**Authors:** Ying‐Chao Han, Bin Ma, Song Guo, Mingjie Yang, Li‐Jun Li, Shan‐Jin Wang, Jun Tan

**Affiliations:** ^1^ Department of Orthopedics Shanghai East Hospital Tongji University School of Medicine Shanghai China; ^2^ Department of Spinal Surgery Shanghai East Hospital Tongji University School of Medicine Shanghai China

**Keywords:** Leptin, Cartilage endplate, Calcification, MAPK, STAT3

## Abstract

Recent findings demonstrate that leptin plays a significant role in chondrocyte and osteoblast differentiation. However, the mechanisms by which leptin acts on cartilage endplate (CEP) cells to give rise to calcification are still unclear. The aim of this study was to evaluate the effects of leptin that induced mineralization of CEP cells *in vitro* and *in vivo*. We constructed a rat model of lumbar disc degeneration and determined that leptin was highly expressed in the presence of CEP calcification. Rat CEP cells treated with or without leptin were used for *in vitro* analysis using RT‐PCR and Western blotting to examine the expression of osteocalcin (OCN) and runt‐related transcription factor 2 (Runx2). Both OCN and Runx2 expression levels were significantly increased in a dose‐ and time‐dependent manner. Leptin activated ERK1/2 and STAT3 phosphorylation in a time‐dependent manner. Inhibition of phosphorylated ERK1/2 using targeted siRNA suppressed leptin‐induced OCN and Runx2 expression and blocked the formation of mineralized nodules in CEP cells. We further demonstrated that exogenous leptin induced matrix mineralization of CEP cells *in vivo*. We suggest that leptin promotes the osteoblastic differentiation of CEP cells *via* the MAPK/ERK signal transduction pathway and may be used to investigate the mechanisms of disc degeneration.

## Introduction

Intervertebral disc degeneration (IDD) is the most important cause of many chronic musculoskeletal disorders of the spine, such as spinal stenosis, disc herniation and lower back pain 1, 2. IDD is a tremendous socio‐economic burden and is among the most common causes of disability and morbidity among workers, in particular, those aged 18–64 years 3, 4, 5, 6. IDD can be caused by various endogenous or exogenous processes, including loading stress 7, inflammation 8, ageing 9, oxidative stress 10 and metabolic stress 11. However, it is universally acknowledged that reduced nutrient supply plays a critical role in disc degeneration 11. Intervertebral discs are the largest avascular structures in the body, and maintenance of nutrient supply is regarded as essential for preventing disc degeneration 12. Nutrients are delivered to the disc primarily by diffusion to and from microvessels in the CEP and the outer annulus fibrosus. The CEP is composed of a thin layer of cortical bone covered by hyaline cartilage produced by chondrocytes. Nutrient supply to the CEP is in turn dependent on vascular supply from surrounding tissues, including the vertebral body 13, 14. CEP degeneration and calcification seriously interferes with nutrient delivery to the disc and is directly related to disc degeneration 15, 16, 17. CEP degeneration is characterized by the loss of cell matrix and calcification of the hyaline cartilage. Importantly, matrix degradation in combination with cessation of cell differentiation leads to a reduction in cartilaginous endplate cells 18.

Leptin is a 16‐kD non‐glycosylated peptide hormone encoded by the obese (ob) gene. It is specifically secreted by white adipose cells, although the placenta, ovaries, skeletal muscles, pituitary, foetal/bone cartilage and human osteoblasts secrete small amounts as well 19, 20, 21, 22, 23. Leptin plays an important role in the regulation of food intake and energy balance, through central signalling in the hypothalamus. Leptin not only has a critical role in the regulation of metabolic processes, but also acts as a proinflammatory cytokine in immune homeostasis, and is involved in the pathogenesis of diseases such as rheumatoid arthritis 24, 25 and psoriasis 26. Recent studies have reported that leptin also regulates inflammation 27, arterial calcification 28 and cartilage damage 29, 30, 31. Leptin levels may also increase during infection, with inflammation enhancing its production through IL‐1β and TNF‐α 32, 33. Some studies have reported leptin receptor expression in adult osteoblasts and chondrocytes, suggesting that leptin signalling is important in bone growth and metabolism 34, 35. Leptin also plays an important physiological role in endochondral bone formation during linear growth, modulating the differentiation and mineralization of chondrocytes in the growth plate 36, 37. Upon binding to its receptor, leptin phosphorylates and activates specific signalling pathways, including mitogen‐activated protein kinase (MAPK) and Janus tyrosine kinase/signal transducer and activator of transcription (JAK/STAT) 37, 38, and plays a critical role in maintaining homeostasis and differentiation of chondrocytes.

Taken together, previous studies suggest that leptin has a close relationship with cartilage differentiation and mineralization. However, the role of leptin in CEP cell ossification has not been fully elucidated. In this study, we investigated whether leptin is induced in rat CEP cell calcification and the underlying mechanism. We constructed a rat model of disc degeneration and found high leptin expression with CEP calcification. We then investigated the stimulatory effects of leptin on CEP cell mineralization *in vitro* and found that leptin promotes the expression of the calcification markers OCN and Runx2 through the activation of MAPK and STAT3 kinases.

## Materials and methods

All animal experiments were performed with the approval and guidance of the Animal Care and Use Committee at Tongji University, which is in accordance with the approved guidelines and principles.

### Animals and surgical procedures

We generated a model of lumbar IDD by modifying the protocol of Wang *et al*. 39. This model has unbalanced dynamics and static force on the lumbar spine, resulting in rapid IDD. Thirty‐six 3‐month‐old male Sprague Dawley rats were randomized into three groups of 12, one to undergo ‘real’ surgery, one to undergo ‘control’ surgery and a third group to be used as a non‐surgical control. In the surgery groups, after a single intraperitoneal injection of 90 mg/kg ketamine‐ anaesthesia of the rats, a midline incision was made over the posterior lumbar spine under intraperitoneal anaesthesia. Using a dorsomedial approach, the paraspinal muscles, spinous process, supraspinous ligaments and interspinous ligaments were removed at each vertebral level. The abdomen and skin were then closed using simple interrupted sutures. In the control surgical group, only a posterior incision was made and then the site was immediately closed. All of the rats were monitored during recovery from anaesthesia. After 9 months, the lumbar spines were harvested for analysis.

Another 36 3‐month‐old male rats were injected weekly with 0.5 mg/kg recombinant rat leptin (R&D Systems, Minneapolis, MN, USA) or phosphate‐buffered saline (PBS) as control. After 6 months, the lumbar spines were harvested for analysis.

### Radiographic analysis of intervertebral disc height

Before surgery and 6 months after surgery, X‐ray radiographs were taken of the experimental and control groups. The average intervertebral disc height of each lumbar spine was calculated by measuring the heights of the anterior, middle and posterior portions of the discs and dividing these values by the average of the adjacent vertebral body heights 40. The percentage disc height was calculated as the average of three discs (L2–L5) from each group.

### Histomorphological staining

Isolated rat spines and adjacent vertebral bodies were fixed in 4% paraformaldehyde, decalcified in ethylenediaminetetraacetic acid (EDTA), embedded in paraffin and sectioned at 4 μm thickness from the mid‐sagittal plane. Sections were stained with either haematoxylin and eosin (H&E; Sigma‐Aldrich, St. Louis, MO, USA) or Alcian Blue 8GX (Sigma‐Aldrich) using standard procedures and photographed at 100× magnification (Nikon Eclipse Ts100; Nikon Instruments, Melville, NY, USA).

### Isolation and culture of CEP cells

Intact lumbar discs were removed by making an incision along the interface between the CEP and vertebral bone, and then, the annulus fibrosus and nucleus pulposus were excised under microscopic visualization. CEP cells were washed with PBS and then minced into small pieces using sterile ophthalmic scissors, after which they were digested with 0.02% collagenase type II (Invitrogen, Waltham, MA, USA) in Dulbecco's modified Eagle medium (Invitrogen) supplemented with 5% foetal bovine serum (FBS; Gibco Langley, OK, USA) at 37°C with shaking overnight. The cell suspension was passed through a sterile nylon mesh filter (70 μm pore size), centrifuged for 10 min. at 2000 r.p.m. and resuspended in Ham's F‐12 medium (F‐12; Invitrogen) with 10% FBS and 1% penicillin/streptomycin (P/S; Invitrogen). Cells were incubated at 37°C, with 5% CO_2_ and 20% O_2_ in a humidified incubator and used for experiments after the second passage of culture following isolation.

### Western blot analysis

Lumbar discs were isolated as described, and following the removal of the annulus fibrosus and nucleus pulposus, proteins were isolated from the remaining CEP tissue using Tissue Protein Extraction Reagent (T‐PER; Thermo Fisher Scientific Waltham, MA, USA, catalog is 78510) stirring at 4°C. For protein extraction from CEP cells, we used radioimmunoprecipitation assay lysis buffer. Proteins were resolved on a 10% SDS‐PAGE gel and transferred to a polyvinylidene difluoride membrane. After blocking with 5% non‐fat‐dried milk in Tris‐buffered saline–Tween (TBST: 10 mM Tris–HCl, pH 8.0; 150 mM NaCl; and 0.5% Tween 20), the membrane was probed overnight at 4°C with primary antibodies. Leptin (ab3583), OCN (ab13418) and Runx2 (ab76956) polyclonal rabbit primary antibodies were supplied by Abcam; and the remaining antibodies, total ERK1/2, phosphorylated ERK1/2 (Thr202/Thr204), total STAT3, phosphorylated STAT3 (Tyr705), total p38 MAPK and phosphorylated p38 MAPK (Thr180/Tyr182) were supplied by Cell Signaling Technology. After washing, a secondary antibody was applied and protein expression was quantified by densitometry analysis using Image J software (NIH, Rockville, Maryland, United States). The densitometry value for each protein was normalized against β‐actin or the non‐phosphorylated form of the protein.

### Immunofluorescence of tissue sections and cells

Leptin‐treated CEP cells were seeded onto glass cover slips, fixed in 4% paraformaldehyde for 30 min. and treated with 0.2% Triton X‐100/PBS for 30 min. Slides were then blocked by incubation with 5% bovine serum albumin (BSA) in PBS for 60 min. Primary antibody was applied (OCN and Runx2) and slides were incubated at 4°C overnight, followed by incubation with DAPI‐conjugated secondary antibody for 1 hr at 37°C. Finally, cells were mounted and visualized using an inverted fluorescence microscope (Nikon Eclipse Ts100; Nikon Instruments). The same protocol was used to immunolabel rat discs following paraffin embedding and sectioning. Images used for comparisons between different treatments were acquired using the same instrument settings and exposure times and were processed equivalently.

### Quantitative real‐time PCR

Total RNA was isolated using a Trizol reagent kit (Invitrogen), and concentration and purity of the RNA were determined spectrophotometrically. A TaKaRa RNA PCR Kit (TaKaRa Bio) was used to prepare cDNA by reverse transcription. Quantitative real‐time PCR (RT‐PCR) assays were performed using a Prism 7500 Fast System (Applied Biosystems, Carlsbad, CA, USA) using the standard curve method. The following gene‐specific primers were used: leptin forward, 5′‐ACTTCATTCCCGGGCTTC‐3′, reverse, 5′‐GGTCTCGCAGGTTCTCCA‐3′; OCN: forward, 5′‐CAGACCTAGCAGACACCATGA‐3′, reverse, 5′‐CTGTGCCGTCCATACTTTCG‐3′; Runx2: forward, 5′‐GCACCATGGTGGAGATCATC‐3′, reverse, 5′‐GTCTGTGCCTTCTTGGTTCC‐3′; β‐actin forward, 5′‐TGAGAGGGAAATCGTGCGTGAC‐3′, reverse, 5′‐AAGAAGGAAGGCTGGAAAAGAG‐3′.

### Transfection with siRNA

Cells at 80% confluence were transfected with either ERK1/2 or STAT3 siRNA (Cell Signaling Technology, Beverly, MA, USA) using Lipofectamine 2000 (Invitrogen), according to the manufacturers' instructions. Cells were lysed 24 hrs after transfection and proteins assayed by Western blotting.

### Alizarin Red and Von Kossa assays

Treated cells were fixed in chilled 70% ethanol for 1 hr, rinsed twice with distilled water (5 min. each wash), stained with Alizarin Red and Von Kossa solutions (GENMED) for 30 min. at room temperature and finally rinsed five times with distilled water. Stained cells were examined using an inverted fluorescent microscope, as previously described.

### Statistical analysis

Data were presented as the mean standard deviation (S.D.) of at least three independent experiments. Statistical Package for the Social Sciences version 17.0 software (SPSS 17.0, SPSS Inc., Chicago, IL, USA) was used for standard statistical analyses. Multiple comparisons of data among the groups were assessed by one‐way analysis of variance (anova) followed by a Fisher's least significant difference test. A *P*‐value<0.05 was considered statistically significant.

## Results

### Lumbar IDD in the rat model

To investigate rat lumbar IDD induced by unbalanced dynamic and static forces, we used X‐ray to measure lumbar disc heights of 3‐month‐old rats prior to and 9 months after surgery.

**Figure 1:**
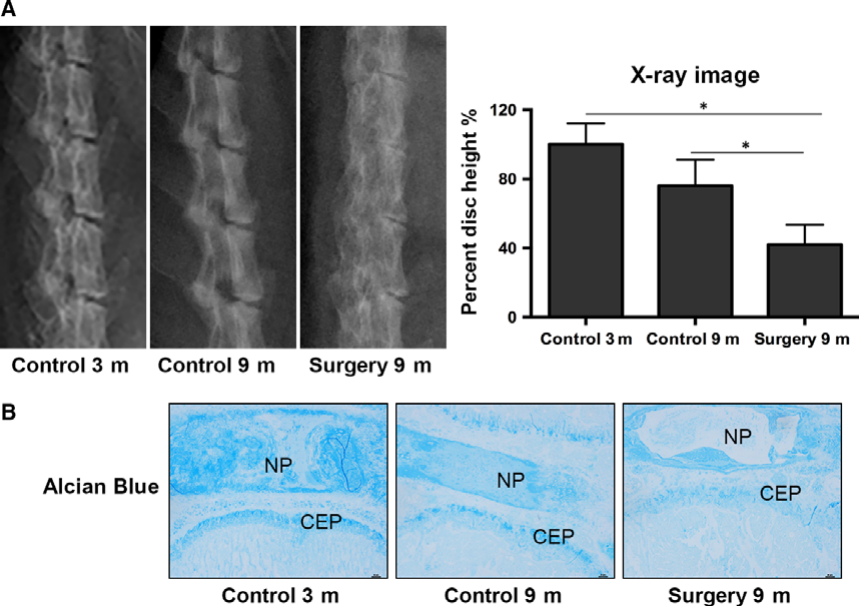
Intervertebral disc degeneration induced by unbalanced dynamics and static forces. (**A**) Lateral radiographs of the rat lumbar spine at 9 months following surgery and the control. The surgery group exhibits lumbar spine degeneration, disc space narrowing and endplate calcification, caused by the unbalanced dynamics and static forces. The lumbar disc height percentage, as determined from measurements made from X‐rays. A significant decrease in the disc height was observed after 9 months in the surgery group compared with the control group. (**B**) Weak Alcian Blue staining in the experimental group compared the control group. Data reported are the average standard error of three discs from at least three rats per group. *P < 0.05, versus Control.

Lateral radiographies of the rat lumbar spine were performed at different time‐points after modelling. Unbalanced dynamic and static forces of lumbar spine caused lumbar spine degeneration and lead to malalignment, disc space narrowing and endplate calcification in surgery groups (Fig. 1A and B). We used Alcian Blue staining to histologically test for the production of matrix proteoglycan; intense staining is visible in the control group, with gradually weaker staining present after surgery (Fig. 1C). This result shows that IDD was significantly worse in the surgery group compared with the non‐surgery group after 9 months. Together, both of these results show that lumbar IDD is present after the surgery.

### Ossification of lumbar CEP cells and increased leptin expression in the rat model

**Figure 2:**
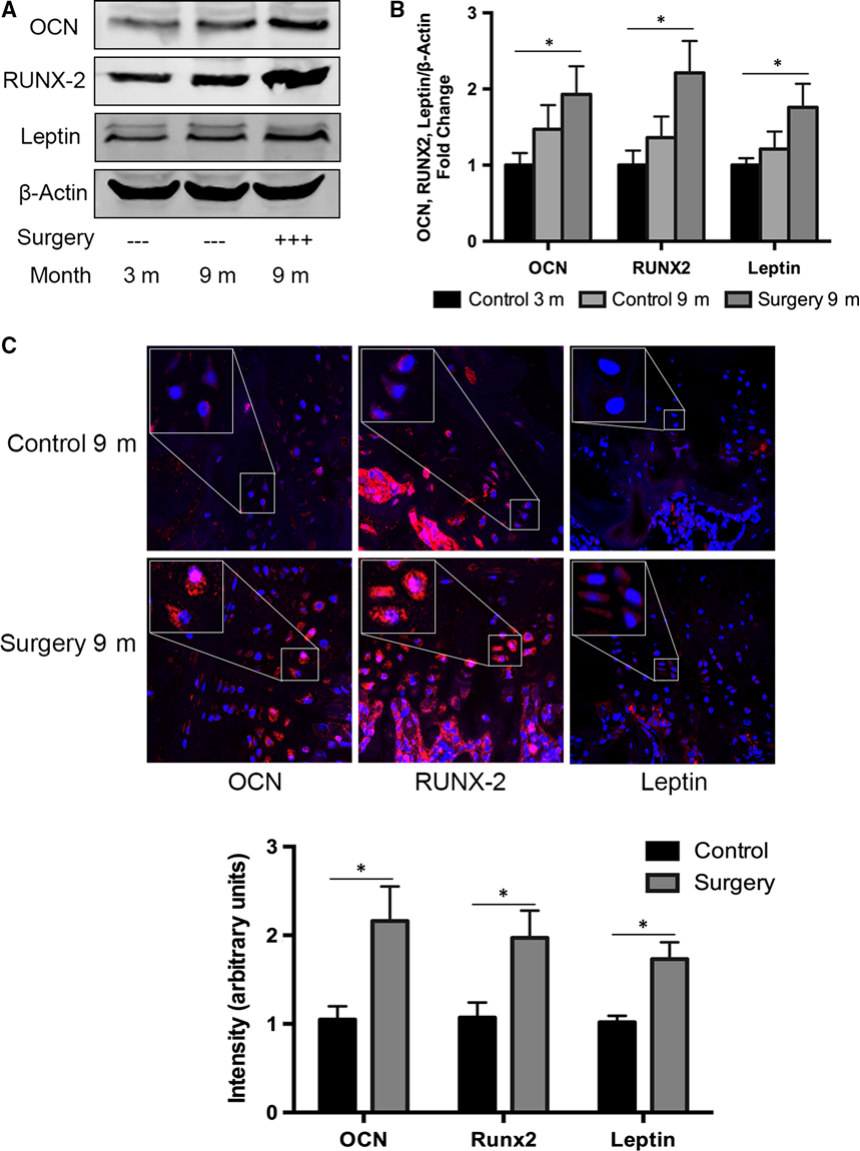
High OCN and Runx2 expression in the Intervertebral disc degeneration rat model. (**A**) Protein levels of leptin, OCN and Runx2, from the intervertebral discs of three‐ and 9‐month‐old rats with or without surgery, as determined using Western blot analysis. CEP protein extracts from 3‐ and 9‐month‐old rats (*n* = 3 for each) were resolved using 10% SDS‐PAGE and probed with leptin, OCN and Runx2 antibodies, using β‐actin as an internal control. (**B**) RNA levels of leptin, OCN and Runx2 in 3‐ and 9‐month‐old rats (*n* = 3 for each) were assayed using quantitative real‐time PCR. Relative units of RNA expression for 3‐month‐old rats were normalized to 1. Asterisks indicate significant differences between treatments (*P* < 0.05). (**C**) Leptin, OCN and Runx2 were detectable in the surgery group and non‐surgery group after 9 months. Scale bar, 25 mm. *P < 0.05, versus Control.

To investigate lumbar CEP degeneration and calcification in the rat model and to investigate the involvement of leptin in disc degeneration and endplate ossification, we examined expression of the cartilage calcification marker genes OCN and Runx2 using Western blotting and quantitative real‐time PCR. Expression of all three markers was elevated in the surgery group after 9 months compared with the control (Fig. 2A and B). To further determine the role of endogenous leptin in maintaining CEP calcification, we performed immunofluorescence assays on lumbar tissue harvested from experimental and control rats 9 months after surgery. Expression levels of leptin, as well as osteoblastic marker genes OCN and Runx2, were significantly higher in tissue isolated from experimental rats than from controls (Fig. 2C). Together, these data show that leptin levels increase in the CEP alongside the accelerated calcification observed histologically in the lumbar disc degeneration process.

### Leptin promotes osteogenic differentiation and mineralization of CEP cells

**Figure 3:**
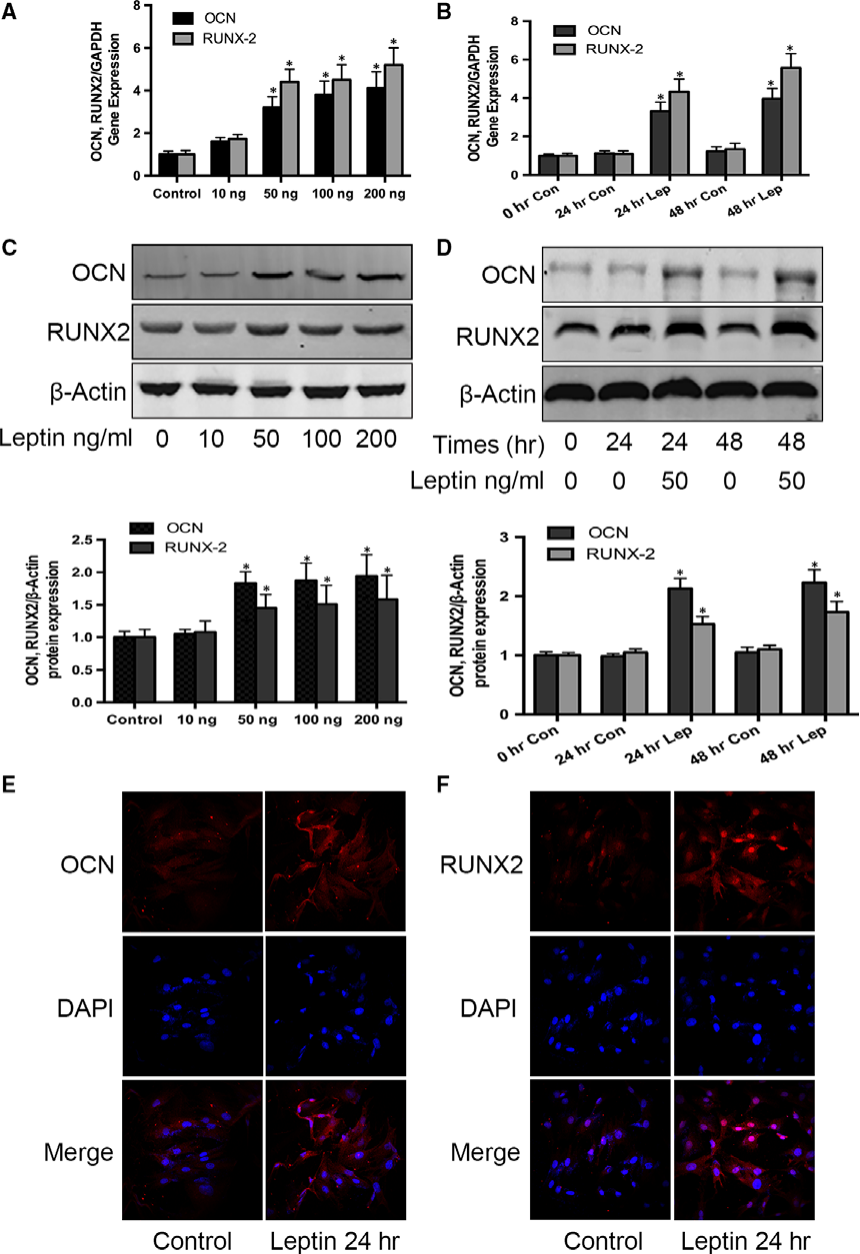
Effect of leptin on OCN and Runx2 expression in CEP cells. Following two passages, primary rat CEP cells were cultured in six‐well plates. Cultured cells were treated with 0, 10, 50, 100 or 200 ng/ml leptin for 48 hrs. RNA levels of OCN and Runx2 were quantified using real‐time PCR (**A,B**) and protein levels quantified using Western blotting and compared to the untreated control with normalization against b‐actin (**C,D**). Cultured cells were also treated with and without 50 ng/ml leptin or OCN for 24 and 48 hrs. OCN and Runx2 protein expression was further determined using immunocytochemistry (**E,F**); blue indicates the nucleus, and red indicates protein expression. Data represent the mean with S.D. of triplicate samples from three independent experiments. Treatments that were significantly different from the 0‐hr controls are indicated with asterisks (*P < 0.05).

Having demonstrated that there is elevated leptin expression and cartilage ossification in IDD rats *in vivo*, we investigated whether leptin plays a role in regulating CEP calcification *in vitro*. CEP cells were treated with increasing concentrations of leptin for 48 hrs, and its effects on the expression of osteogenic markers OCN and Runx2 were examined using quantitative RT‐PCR. With increasing concentrations of leptin, both OCN and Runx2 expression levels gradually increased; up‐regulation was detectable at 50 ng/ml leptin, and maximal up‐regulation was measured at 200 ng/ml (Fig. 3A). This result shows that leptin induces OCN and Runx2 expression in a dose‐dependent manner. To confirm the regulatory effect of leptin on osteogenic gene expression levels, we also measured the expression of OCN and Runx2 in CEP cells after treatment with leptin for 24 and 48 hrs. Significantly increased expression levels of both OCN and Runx2 were detected in the leptin‐treated cells at both time‐points in comparison with controls (Fig. 3B).

To further support the RT‐PCR results, we investigated whether the measured increases in OCN and Runx2 RNA expression correspond with increased protein expression. Following treatment of CEP cells with different concentrations of leptin for 48 hrs, Western blotting revealed that 50, 100 and 200 ng/ml leptin significantly promoted OCN and Runx2 protein expression, while 10 ng/ml leptin had no significant effect (Fig. 3C). We then measured OCN and Runx2 protein expression in CEP cells following treatment with or without 50 ng/ml leptin for 24 and 48 hrs. At both time‐points, OCN and Runx2 expression increased following leptin treatment (Fig. 3D). No changes were observed in the expression levels of the β‐actin housekeeping protein between any time‐points or treatments. Immunocytochemical staining was used to further confirm the changes in OCN and Runx2 protein expression levels in response to leptin exposure (Fig. 3E and F). These data suggest that leptin up‐regulates the osteogenic markers OCN and Runx2, thereby promoting spontaneous osteoblastic differentiation of CEP cells.

### Leptin induces osteogenic differentiation in CEP cells through the ERK/STAT3 signalling pathway

**Figure 4:**
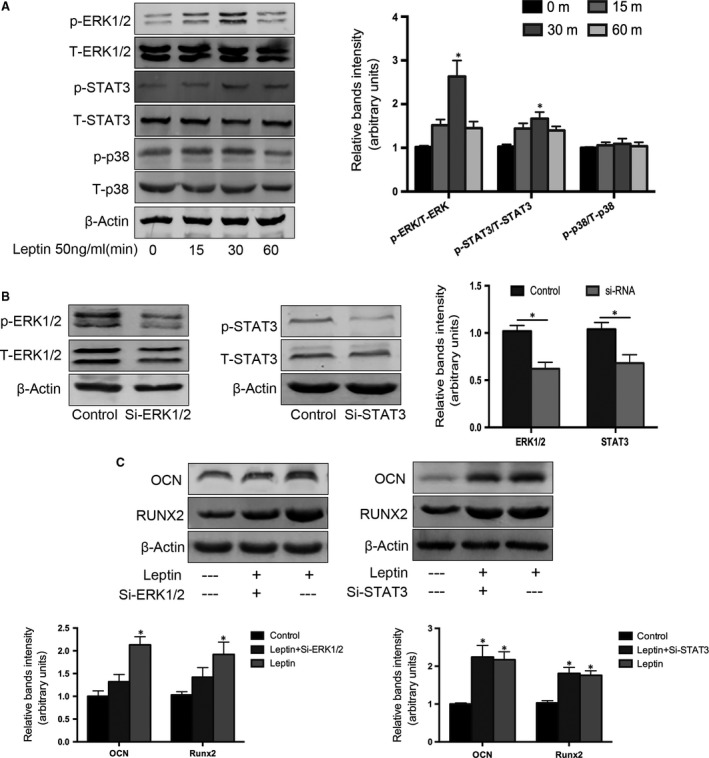
Effect of leptin on MAPK/STAT3 phosphorylation in CEP cells. (**A**) CEP cells were cultured in serum‐free media for 1 hr and then treated with or without 50 ng/ml leptin over indicated time periods. To examine MAPK and STAT3 pathway activation, cell extracts were analysed by Western blotting using antibodies against the phosphorylated forms of MAPK and STAT3 pathway proteins. ERK1/2 and STAT3 were maximally phosphorylated 30 min. after leptin treatment; however, p38 phosphorylation did not change. (**B**) CEP cells were treated separately with siRNAs targeted to ERK1/2 (si‐ERK1/2) and STAT3 (si‐STAT3) that inhibit the phosphorylation of ERK1/2 and STAT3. (**C**) CEP cells were incubated with si‐ERK1/2 or si‐STAT3 for 48 hrs and then treated with 50 ng/ml leptin for 24 hrs, after which OCN and Runx2 protein expression levels were measured by Western blot analysis. Data represent the mean ± S.D. of triplicate samples from three independent experiments. Treatments that were significantly different from the 0‐hr controls are indicated with asterisks (*P < 0.05, versus Control).

Mitogen‐activated protein kinases are a family of kinases that transduce signals in response to a wide range of stimuli 41, 42. MAPKs have been demonstrated to contribute variously to cellular events, including cell proliferation 43, differentiation 44, migration 45 and apoptosis 46. To identify whether MAPKs play a role in the osteogenic differentiation response of CEP cells, we evaluated the phosphorylation of three MAPKs, ERK1/2, STAT3 and p38, using Western blot analysis. Treatment of CEP cells with 50 ng/ml leptin induced rapid and transient phosphorylation of ERK1/2 and STAT3. Both MAPKS were detectable from 15 min. after treatment, had maximal phosphorylation at 30 min. and then gradually declined to pre‐treatment levels by 60 min. However, p38 phosphorylation was not altered in a time‐dependent manner in response to leptin treatment of CEP cells. Unlike the phosphorylation levels of ERK1/2 and STAT3, the total ERK1/2, STAT3 and β‐actin levels remained unchanged between samples (Fig. 4A). Next, we showed that transfection of CEP cells with ERK1/2‐ or STAT3‐specific siRNA significantly inhibited leptin‐induced ERK1/2 and STAT3 phosphorylation (Fig. 4B). To further investigate whether leptin‐induced OCN and Runx2 expression levels are mediated through the ERK or STAT3 pathways, CEP cells were transfected with ERK1/2 or STAT3 siRNA first and then treated with 50 ng/ml leptin for 48 hrs. After Western blot analysis, we found that ERK1/2 siRNA transfection blocked leptin‐induced elevations in OCN and Runx2 protein. However, STAT3 siRNA transfection prior to leptin treatment had no effect on OCN or Runx2 expression.

**Figure 5:**
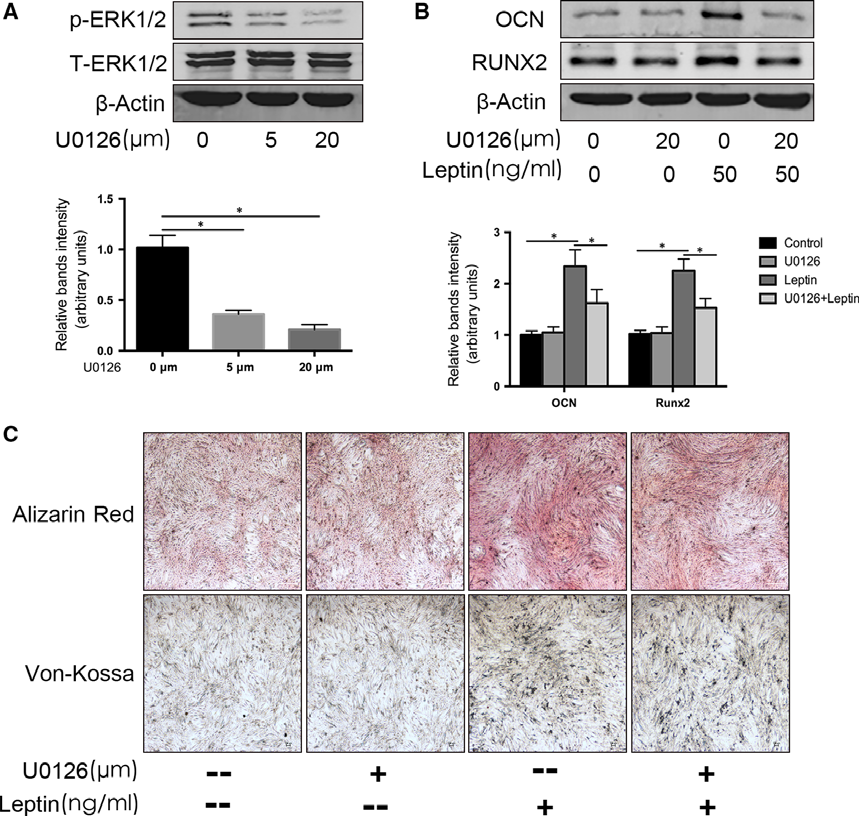
Leptin induces osteoblastic differentiation and mineralization of CEP cells through the MAPK/ERK1/2 signalling pathway. (**A**) Cultured CEP cells were treated with 5 or 20 μM U0126 for 60 min. and then induced with leptin for 30 min., after which phosphorylated ERK1/2 (p‐ERK1/2) and total ERK1/2 protein levels were analysed by Western blot analysis. (**B**) The CEP cells were pre‐treated with 5 or 20 μM U0126 for 60 min., followed by treatment with 50 ng/ml leptin for 48 hrs, after which OCN and Runx2 protein expression levels were measured by Western blot analysis. (**C**) CEP cells incubated with 50 ng/ml leptin for 14 days were stained with Alizarin Red and Von Kossa solutions to detect mineralization (original magnification, 40×). Data represent the mean ± S.D. of triplicate samples from three independent experiments (*P < 0.05, versus Control).

Additional experiments were carried out to further confirm the role of ERK1/2 phosphorylation in leptin‐induced OCN and Runx2 expression, using the ERK1/2 phosphorylation inhibitor U0126 (Cell Signaling Technology). U0126 dose dependently suppressed the effect of leptin on ERK1/2 phosphorylation in CEP cells (Fig. 5A). Moreover, treatment of CEP cells with 20 μM U0126 for 30 min., followed by treatment with 50 ng/ml leptin for 48 hrs, blocked the leptin‐induced up‐regulation of OCN and Runx2 protein expression (Fig. 5B).

We also used an Alizarin Red assay to investigate CEP cell matrix mineralization, which is a marker for osteoblastic differentiation. CEP cells incubated with 50 ng/ml leptin for 14 days showed significantly increased Alizarin Red staining; however, cells pre‐treated with U0126 prior to leptin treatment showed decreased staining (Fig. 5C). Taken together, these data strongly suggest that leptin‐induced CEP cell calcification is mediated by ERK1/2 phosphorylation.

### Exogenous leptin induces elevated OCN and Runx2 expression in the rat IDD model without significant changes to disc histomorphology

**Figure 6:**
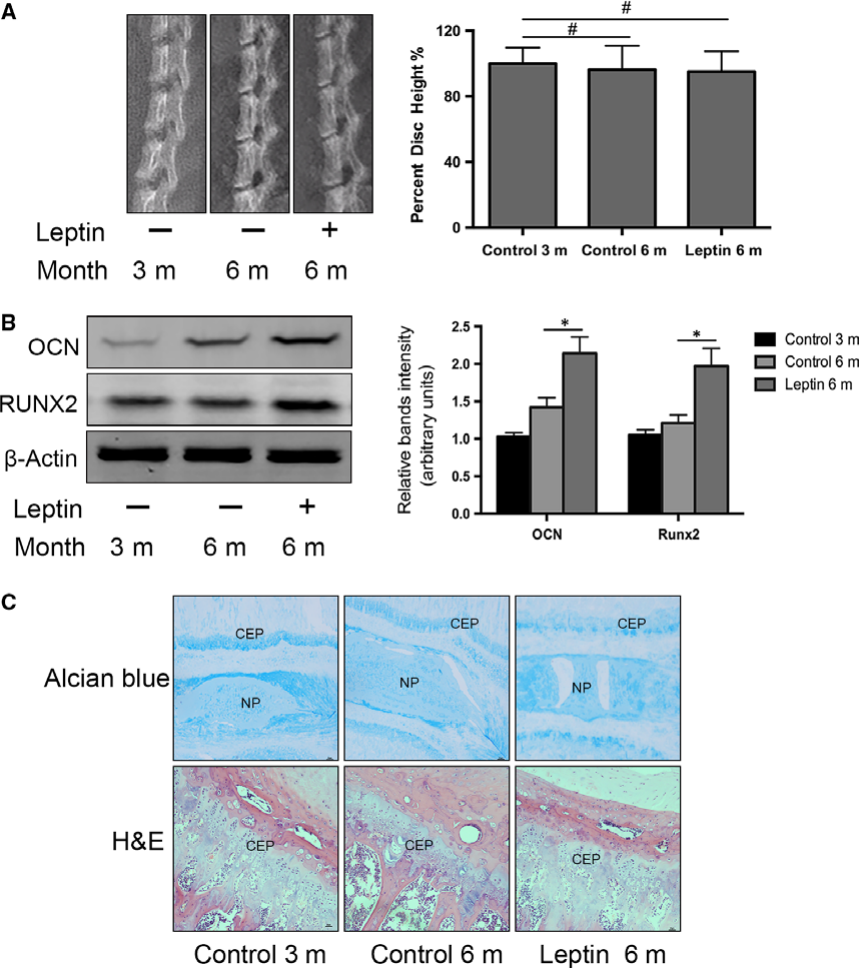
Rat intraperitoneal injection of exogenous leptin improves CEP matrix mineralization in the rat Intervertebral disc degeneration model. (**A**) Western blot analysis of leptin, OCN and Runx2 protein levels in 3‐ and 6‐month‐old rats with or without leptin injections (*n* = 3 for each treatment). (**B**) X‐ray radiographs showing that the percentage of lumbar disc heights did not change after 6 months of leptin injections in comparison with the control group. (**C**) H&E and Alcian Blue staining comparisons of the 3‐ and 6‐month‐old rats with or without the leptin injections, showing reduced cartilage matrix and cartilage cells. Error bars represent the standard error of three discs from at least three rats per treatment group (*P < 0.05, versus Control).

To further explore the physiological role of exogenous leptin in rat CEP ossification, we intraperitoneally injected 3‐month‐old rats with recombinant rat leptin (0.2 mg/kg per week). After 6 months, rat spines were isolated for CEP protein extraction, X‐ray analysis and histological staining (Fig. 6). Western blot analyses of CEP proteins revealed increased OCN and Runx2 expression following 6 months of leptin injections in comparison with the control group (Fig. 6A). However, X‐ray analysis and histological staining found that the morphologies of the intervertebral discs and cartilage were not significantly different between the experimental and control groups (Fig. 6B and C).

## Discussion

Previous studies have demonstrated that leptin not only regulates food intake and metabolic rate, but also modulates bone formation and metabolism 47, 48, 49. In our *in vivo* experiments, we measured elevated leptin in the degenerating discs of surgically treated rats, and this increased leptin correlated with increased CEP osteoblastic differentiation. Our *in vitro* analyses revealed that rat CEP cells up‐regulate expression of the osteogenic markers, OCN and Runx2, *via* the ERK1/2 signalling pathways. To the best of our knowledge, this research is the first showing the effects of leptin on the mineralization potential of disc CEP cells.

Recently, numerous investigations have suggested that leptin plays a critical role in the pathophysiology of diseases such as diabetes 50, lipodystrophy 51 and cardiovascular disease 52. Studies have also reported that obesity is a risk factor for vascular calcification 53, 54, 55 and arthritis 56, 57. We have previously observed that leptin expression is elevated in ob patients 58, suggesting that leptin might play an important role in the development of certain vascular and bone diseases. Some studies have demonstrated that there are increased leptin receptors in human osteoblasts and chondrocytes, and binding with the leptin receptor directly affects bone growth and metabolism 34, 48. It has also been reported that leptin is secreted in cartilaginous skeletal growth centres, articular cartilage and in osteoblasts and that high levels of leptin may damage cartilage 23, 59. In this study, to explore the relationship between leptin and disc CEP ossification, we first constructed a rat disc degeneration model, following the methods of Wang *et al*. 39. We chose to use 3‐month‐old rats for the surgery, because rats reach approximately 90% skeletal maturity by this age 60. We found that 6 months following surgery was sufficient time for the lumbar disc degeneration model to develop and present CEP ossification. From X‐ray and Alcian Blue analysis, we detected lumbar misalignment and loss of the cartilaginous matrix, respectively. During cartilage calcification, expression levels of many bone‐related factors increased, including OCN, Runx2, osteoprotegerin and alkaline phosphatase. Previous studies have also shown that OCN 61, Runx2 62 and Sox9 63 play critical roles in bone metabolism, energy expenditure and chondrogenic differentiation. Other studies have found that insulin‐like growth factor 1 64, transforming growth factor‐β 65 and fibroblast growth factor 23 35 are up‐regulated by leptin and impact bone growth. In our experiments, Western blotting and immunofluorescence data show a steady increase in OCN and Runx2, as well as leptin expression, in disc CEP cells of the surgery group compared with the control group. Therefore, we suggested that CEP calcification is caused by elevated leptin expression in the degenerating disc.

To further demonstrate that leptin promoted the osteoblastic differentiation and mineralization of CEP cells, we isolated and cultured rat CEP cells. Cells were treated with different concentrations of leptin over different periods of time. RNA and protein expression level measurements of the osteoblastic differentiation markers OCN and Runx2 showed that leptin can induce their elevated expression in a dose‐ and time‐dependent manner. Moreover, we found that 50 ng/ml leptin significantly increased OCN and Runx2 expression. Taken together, these data suggest that leptin is an important promoter of osteoblastic differentiation and mineralization in CEP cells.

To investigate the molecular mechanism by which leptin affects osteoblastic differentiation, we evaluated signalling pathways that are known to be associated with calcification and differentiation. Numerous studies have demonstrated that leptin activated the MAPK signalling pathway 66, 67. It has also been shown that the MAPK pathway is involved in osteoblastic differentiation and mineralization and mediates leptin signalling in chondrocytes 56, 68 and vascular smooth muscle cells 69, 70, 71. MAPK kinase is a heterogeneous kinase family that phosphorylates specific amino acid residues; the extracellular signal‐regulated kinases and the p38 kinases are important members of this family. In the present study, we investigated whether leptin induced the activation of the ERK1/2 signalling pathway in CEP cells. Some studies have reported that p38 MAPKs are activated during chondrogenesis and in endochondral ossification 72; however, in our experiments, we did not find any significant effect of leptin on p38 phosphorylation. This suggests that p38 is not a major MAPK pathway involved in leptin‐induced differentiation in CEP cells.

STAT3 is thought to play a critical role during cell survival, proliferation and differentiation 73 and acts as a major transcription factor. STAT3 interacts with other nuclear factors associated with leptin signalling that regulate transcription of target genes 74. It has been reported that STAT3 not only regulates chondrocyte differentiation 75, but also modulates the increased expression of matrix metalloproteinases in response to leptin stimulation 76. We found that leptin activated STAT3 in a time‐dependent manner, consistent with previous reports.

To further characterize the importance of ERK1/2 and STAT3 signalling pathways in leptin‐induced CEP cell calcification and differentiation, we transfected cells with siRNAs to silence ERK1/2 and STAT3 phosphorylation. After silencing of ERK1/2 phosphorylation, OCN and Runx2 were no longer up‐regulated in response to leptin. However, silencing of STAT3 phosphorylation had no effect on OCN and Runx2 expression. This result suggests that although leptin activates STAT3, the STAT3 pathway probably does not influence OCN and Runx2 expression. To further confirm that the ERK1/2 signalling pathway plays an essential role in the osteoblastic differentiation and mineralization of CEP cells, we showed that the ERK1/2 inhibitor U0126 inhibited the leptin‐induced up‐regulation of OCN and Runx2 protein expression and reduced cartilage nodules.

Peritoneal injection of rat recombinant leptin increased the leptin concentration in rat serum. After 6 months of weekly injections, we found that exogenous leptin injections did not result in abnormal bony tissue formation or degeneration in the lumbar disc. The intervertebral disc is an avascular structure; therefore, despite the elevated leptin concentration in the serum, there may not have been adequate diffusion to the disc to elicit morphological changes. However, at the molecular level, increased OCN and Runx2 expression levels were detected in isolated CEP protein, suggesting that exogenous leptin injections can induce CEP calcification.

In conclusion, this study has demonstrated that disc degeneration is associated with elevated leptin expression, and leptin promotes the osteoblastic differentiation and mineralization of lumbar CEP cells. We also found that leptin induces the activation of the ERK1/2 and STAT3 signalling pathways, which leads to calcification of CEP cells. This study has significant implications for understanding the mechanisms involved in CEP calcification. Nevertheless, CEP degeneration and ossification is complex and involves numerous contributing factors; consequently, further research will be needed to confirm our findings and fully characterize this process.

## Author contributions

J.T. and S.J.W. designed the study. Y.C.H. and B.M. developed the methodology. Y.C.H. and S.J.W. performed the experiment. B.M. and L.J.L. collected the data. Y.C.H. and J.T. performed the analysis. Y.C.H. and S.J.W. wrote the manuscript. All authors reviewed the manuscript.

## Conflict of interest

The authors confirm that there are no conflicts of interest.
